# Mechanisms for log normal concentration distributions in the environment

**DOI:** 10.1038/s41598-021-96010-6

**Published:** 2021-08-12

**Authors:** August Andersson

**Affiliations:** Department of Environmental Science and the Bolin Centre for Climate Research, Stockholm University, 10691 Stockholm, Sweden

**Keywords:** Biochemistry, Biogeochemistry, Climate sciences, Ecology, Environmental sciences, Hydrology, Limnology, Natural hazards, Ocean sciences, Solid Earth sciences

## Abstract

Log normal-like concentration distributions are ubiquitously observed in the environment. However, the mechanistic origins are not well understood. In this paper, we show that first order exponential kinetics onsets log-normal concentration distributions, under certain assumptions. Given the ubiquity of exponential kinetics, e.g., source and sink processes, this model suggests an explanation for the frequent observation in the environment, and elsewhere. We compare this model to other mechanisms affecting concentration distributions, e.g., source mixing. Finally, we discuss possible implications for data analysis and modelling, e.g., log-normal rates and fluxes.

## Introduction

**Figure 1:**
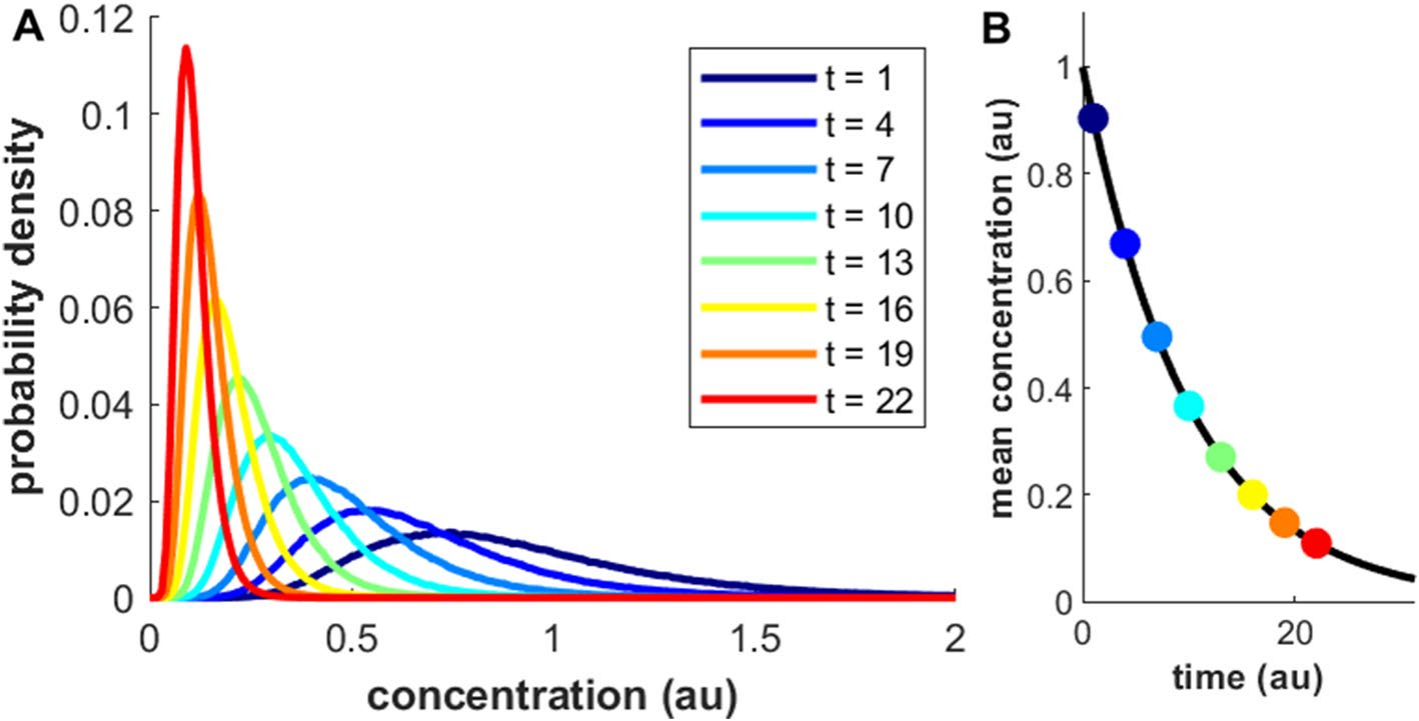
Illustration of the temporal evolution of a log-normal concentration distribution during continuous kinetic degradation. Panel (**A**): Concentration distributions depicted at different times (t = time; Eqs. 3 and 4a–b). Panel (**B**): Temporal evolution of the corresponding mean concentrations, displaying exponential decay (Eq. 5).

Log normal-like distributions are observed across scientific disciplines, including Earth and Environmental Sciences (Fig. 1A, Eq. 3)^1–4^. A fundamental question is how this dependency emerges. In the fields of Ecology and Economics, mathematical models for economic or population growth predicts log-normal distribution onset^3,5^. For environmental systems, a few studies discuss general mechanisms for the emergence of log-normal concentration distributions^6–8^. These studies are intuitively appealing, but with less theoretical rigor, while the underlying physico-chemical mechanisms remains largely unclear.

Ascribing empirical data to a given parametric distribution function, such as the log-normal distribution, is often advantageous for statistical analysis^9–11^. Much efforts have been dedicated towards such statistical analysis of environmental data. However, with a number of important exceptions, less interest has been directed towards understanding the underlying scientific question: what physico-chemical mechanisms drive the onset of different observational distributions, e.g., the log-normal distribution^6–8,12,13^?

An example of a physical–chemical mechanism governing the onset of the normal concentration distribution is the mixing of multiple sources. For instance, consider emissions of carbon monoxide (CO) from many chimneys across a village. As the emission flux from each chimney vary randomly over time, the total concentration becomes the sum of many stochastic variables, and by the Central Limit Theorem the distribution tends to normality. Even though virtually any component in the environment is the sum of a number of different sources, much of environmental data display distinctively non-normal distributions, including examples with CO^9–11,14^. This suggests that there must be other mechanisms—mechanisms that to some extent override the source mixing mechanisms—pushing towards other concentration distributions. One such mechanism must then be the driver towards log normal-like distributions in the environment. What physical–chemical mechanism(s) may predict the onset of a log-normal dependence, as well as explain the comparable ubiquity in the environment?

In this paper, we investigate the relationship between first order kinetics and log-normal concentration distributions in environmental systems (Fig. 1A–B). A key concept in this regard is stochastic variability. To incorporate random fluctuations, we formulate a kinetic model as a stochastic differential equation (SDE). For clarity, we develop the model for the specific case of a kinetic sink process. However, we emphasize the general applicability of this mathematical framework to any process exhibiting first order kinetics, e.g., chemical formation or partitioning kinetics, also beyond environmental systems. While the mathematical treatment (see SI for details) provides rigor, we highlight physical interpretation: exponential kinetics is a possible mechanism that induces log-normal concentration distributions in the environment. This mechanism is compared with other processes that influence concentration distributions. Finally, we discuss a number of implications for data analysis and modelling.

## Theory

Consider a component, X, in an environmental system, e.g., an organic pollutant, soot particles or CO. The sink or loss kinetics is often described by a first-order differential equation, with sink rate *k*:

1 $$\frac{d\left[ X \right]}{{dt}} = - k\left[ X \right]$$

This relation results in an exponentially decaying temporal dependence (Fig. 1B, Eq. 5). For a few systems, such as radioactive decay, this description captures the dynamics well, in the sense that *k* is a constant. However, for many environmental systems, *k* is dependent on, e.g., state variables such as temperature and pressure, but also on the concentrations of other components, since sinks often involve chemical reactions. Thus, *k* is commonly a function of time.

As an example, the chemical breakdown of CO in the atmosphere takes place through the reaction with the hydroxyl radical (OH), and the reaction rate thus varies in time with the OH concentration^14^. OH in the atmosphere is formed through photo-chemical reactions, and the abundance thus depends on, e.g., the diurnal variations in sunlight. In addition to such deterministic dependence, the OH concentration typically also displays a near-random variability over time, reflecting the fluctuations of many parameters (e.g., chemical reactants, humidity, turbulent diffusion, temperature or cloudiness). Consequently, the CO sink rate therefore also displays stochastic variability.

Overall, randomly varying rates are expected to be common in various environmental systems, including many different types of processes, e.g., chemical reactions (e.g., CO with OH), deposition, photo-degradation or kinetic partitioning. In general, we can write the rate *k* as the sum of a deterministic and a stochastic part. Here, we are mainly interested in understanding the impact of the stochastic part, and therefore for simplicity assume that the deterministic component is a constant (μ_k_). We may re-write Eq. (1) in the form of a stochastic differential equation (SDE):

2 $$\frac{d\left[ X \right]}{{dt}} = - \left( {\mu_{k} + \sigma_{k} \eta \left( t \right)} \right)\left[ X \right]$$

where μ_k_ is the mean reaction rate and σ_k_ is the magnitude of the stochastic fluctuation. The function η(t) describes the time-dependency of the random fluctuations (with amplitude 1), which we here assume is independent and identically (i.i.d.) normal distributed.

Since η(t) fluctuates randomly at each time point, the solutions to Eq. (2) will also fluctuate randomly, and we obtain a different solution (or path) when solving it at different instances. However, our present goal is not to generate different realizations, but to evaluate the overall behavior: to obtain the concentration distribution, i.e., the probability density distribution (pdf) of the different realizations. The equation that describes the temporal evolution of the pdf is the Fokker–Planck equation (FPE), which can be derived from a corresponding SDE^15^. The FPE is a (non-stochastic) partial differential equation, and the solution is a pdf with two variables: the concentration of X and time (Fig. 1A). The different steps involved in obtaining the FPE that corresponds Eq. (2) and then solving it, are provided in the SI (S1 text).

The solution of the FPE corresponding to Eq. (2) is the log-normal distribution, with parameters μ and σ, of the form (Fig. 1A):

3 $$P\left( {\left[ X \right]} \right) = \frac{1}{{x\sqrt {2\pi \sigma^{2} } }}e^{{ - \frac{{\left( {\ln \left( {\left[ X \right]} \right) - \mu } \right)^{2} }}{{2\sigma^{2} }}}}$$

Thus, a generic kinetic sink model predicts the onset of log-normal variability, under the assumption of a randomly fluctuating sink rate.

If we now, instead of a sink process, consider the formation of a reaction product. For example, the formation of CO_2_ from the reaction of CO with OH: what concentration distribution do we expect for CO_2_? In the supplementary materials (S2 text) we show that a randomly fluctuating formation rate induces log-normal concentration distributions in reaction product as well. Taken together, we thus find that both source and sink kinetics may lead to log-normal concentration distributions. In fact, any first order kinetics process with a stochastic rate of the form of Eq. (2) will induce log-normal dependence.

Equation (3) is a generic expression for the log-normal distribution, and thus provides no specific information regarding the temporal kinetics associated with the system in Eq. (2). Following our treatment in the SI (S1 text), we can express the temporal evolution of the concentration distribution of X in Eq. (3), with the following parameters (Fig. 1A, Eq. S21):

4a $$\mu = \mu_{0} - \left( {\mu_{k} + 0.5\sigma_{k}^{2} } \right)t$$

4b $$\sigma^{2} = \sigma_{0}^{2} + \sigma_{k}^{2} t$$

The parameters μ_0_ and σ_0_ represent the concentration distribution of X at t = 0.

It is instructive to examine the temporal evolution of the mean (*m*), which for a log-normal distribution is given by (here, *k* = μ_k_, since the mean of η(t) is zero):

5 $$m = \exp \left( {\mu + 0.5\sigma^{2} } \right) = [X\left( 0 \right)]e^{ - kt}$$

The mean temporal dependence is thus recognized as the regular exponential solution to Eq. (1), where X(0) (= exp(μ_0_ + 0.5σ^2^_0_)) is the mean concentration of X at t = 0 (Fig. 1B).

## Results and discussion

### Log normal concentrations in the environment

Empirical concentration distributions in the environment exhibit a wide variability, ranging from familiar parametric forms, e.g., normal and log-normal distributions, to more complex shapes^9–12^. Overall, the concentration distribution reflects the different processes that govern environmental fate, e.g., emissions/formation, transport processes, source mixing, sinks and dependences on other variables and processes. In principle, we may therefore extract mechanistic insights into the lifecycle of an environmental component from analysis of the concentration distribution. Although such deconvolution is intangible in the general case, there are specific cases where there are known mechanistic origins for certain distributions. For instance, source mixing in the environment will tend the distribution towards normal.

In this paper we present a model for how log-normal distributions may emerge in the environment (Fig. 1A). This topic has been addressed in a few earlier studies^6–8^. A joint argument in these is the implication of the ‘Multiplicative Central Limit Theorem’ (or ‘Gibrat’s law’), which applies to processes that include the product of many random variables. If we take the logarithm of the product of many random variables, we have the sum of many random variables, by which the Central Limit Theorem applies, which predict a normal distribution. Back-transforming the logarithm, yields a log-normal distribution. Although this heuristic argumentation does not provide specific physico/chemical mechanisms as to how the multiplication of multiple random variables may commonly appear in the environment, it does provide an intuitive framework.

The present contribution is based on a physico/chemical first order exponential kinetics model (Fig. 1B). Starting with an ordinary differential equation (Eq. 1), we introduce a stochastically variable rate, resulting in a stochastic differential equation (Eq. 2). By solving the corresponding Fokker–Planck equation (FPE) (see Theory section and SI for details) we derive the log-normal distribution (Eq. 3), under certain assumptions about stochastic variability. Since exponential kinetics are commonly observed for many processes in the environment, this model provides a potential explanation for the relative ubiquity of lognormal distributions in the environment.

Observations of lognormal-like concentration distributions in the environment include a wide array of components (e.g., aerosols; bulk organic carbon; heavy metals; organic pollutants; minerals; trace gases; inorganic ions; humic matter; biomarkers; radionuclides; microplastics; pharmaceuticals and pesticides) in various environmental sub-compartments (e.g., groundwater; watersheds; urban air; precipitation; rocks; stormwater; indoor air; soils; wastewater; marine sediments; landfills; lake sediments; peat; glaciers; background air; biota and humans)^2,3,12–14,16–38^. However, even though log normality is relatively common in the environment, we emphasize that it by no means is generally observed^10–12^: see discussion in the next section.

Ascribing a data set to a specific parametric form, e.g., log-normal, may provide mechanistic insights, but also typically simplifies statistical treatment^11^. Among multiple approaches, a straight-forward approach to test for log-normality is by log-transforming the data and then use one of the many well-established tests for normality, e.g., the Shapiro-Wilks test^9,39,40^. Specific aspects of analysis of log-normal data sets, e.g., the impact of non-detects or data averaging, or data sampling strategies, have been discussed in some detail in the literature^12,41–43^. However, even though we may attribute a certain data set to a parametric distribution, there are many empirical situations where the data appears to follow a complex/un-known distribution. Can we identify some general principles that may help disentangle observed variability, even beyond common parametric forms?

### Three mechanisms influencing concentration distributions

Any environmental system is governed by a multitude of different processes, occurring simultaneously. Fortunately, some mechanisms appears to be more prevalent than others, and we can make simplifications, allowing modeling, e.g., the currently presented model for log-normal dependence. To attempt a more general description for concentration distributions, it may prove useful to consider a few different principal mechanisms. Here we explore three such mechanisms:

#### Kinetics

**Figure 2:**
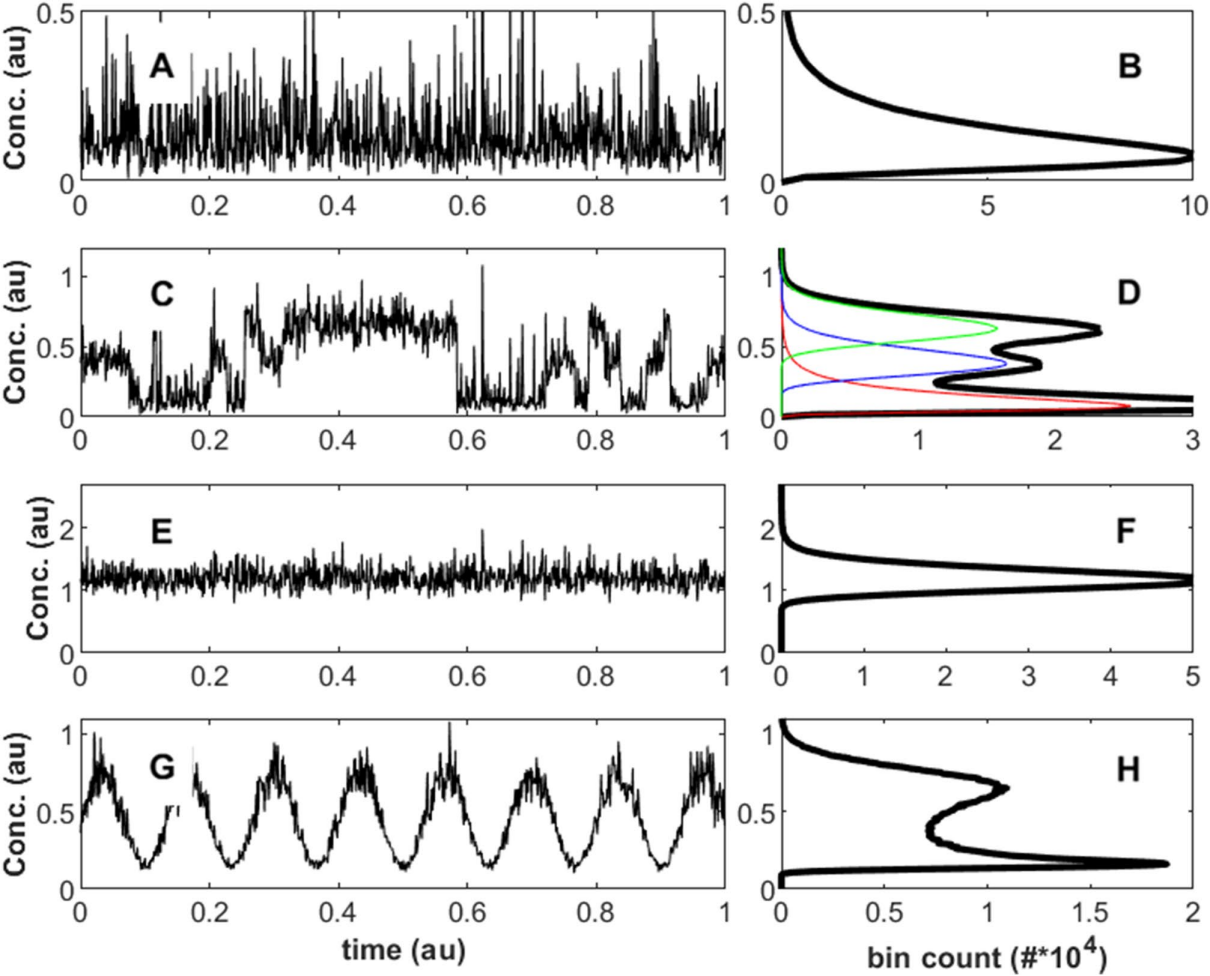
Numerical simulations (random sampling) of time-series concentration data and corresponding distributions, illustrating how different processes may influence concentration distributions. Panels (**A**)–(**B**): Log normally-distributed data (Eq. 3), e.g., influenced by exponential kinetics. Panels (**C**)–(**D**): Data reflecting the random fluctuation between three different log-normally distributed states (green, blue and red), yielding a multi-modal (mixture) distribution. Panels (**E**)–(**F**): Random mixing of the three distributions from panels (**C**)–(**D**), yielding a convolution distribution, tending towards a normal distribution. Panels (**G**)–(**H**): The log-normal distribution of panels (**A**)–(**B**), modulated by an oscillatory function (here sine function), illustrating the impact of, e.g. diurnal or seasonal cycles on observed distributions. The simulations were conducted using Matlab (ver. R2019b).

The model presented here is a common first order approximation of kinetics, e.g., of source and sink processes (Fig. 2A–B). In reality, dynamical systems may be highly complex, beyond the present formulation with a constant deterministic part (μ_k_, Eq. 2), with multiple coupled components, non-linearities, feedbacks and heterogenous interactions. How such more complex kinetics influence concentration distributions is a topic for further scientific discovery.

#### Mixing

The concentration of a component in the environment is typically the sum of emissions from many sources. If these combine randomly, the concentration distribution approach normality (Fig. 2E–F). However, this type represents one limit of mixing—where the resulting distribution at each time point is the sum distribution (convolution) of many variables. Another limit is where the contributions from different sources are separated, such that the concentration at any given measurement point (e.g., time point) reflects one individual source (Fig. 2C–D). An example could be an atmospheric measurement site located between two cities: by wind direction each time point is mainly dominated by either city. In this limit the concentration data will tend to a multi-modal (or ‘mixture’) distribution.

#### External variables

In the model described in the Theory section the non-stochastic part of Eq. (2) is a constant (μ_k_). But this parameter may also vary with external parameters, e.g., state variables. For instance, diurnal or seasonal variations of temperature, pressure or sunlight, including phase transitions, may strongly influence concentration distributions (Fig. 2G–H).

These three classes of mechanisms influence the lifecycle of a component in the environment, but their relative importance regarding how concentration distributions are affected may be highly variable. For certain instances it may be possible to ascribe a certain parametric function to the data, e.g., a normal distribution for well-mixed sources or a log-normal suggesting a kinetic domain. However, for situations with more complex, multi-modal or broad distributions both statistical treatment and interpretations are more challenging. For instance, quite similarly looking distributions may emerge from rather different mechanisms, e.g., dependence on an externally oscillating parameter or a stochastically jumping mixture distribution (e.g., compare Fig. 2C–D with Fig. 2G–H).

### Outlook

The shape of empirical concentration distributions is determined by the lifecycle dynamics and thereby contain information of environmental fate. In this paper we show that log-normality suggests influence by first order exponential kinetics (Eqs. 1 and 5; Fig. 1A–B). Reflecting the overall non-equilibrium state of environmental systems, there are a number of different processes/mechanisms that may exhibit exponential kinetics and thereby drive concentrations towards log-normality, e.g., emissions/chemical formation (e.g., of primary or secondary pollutants), degradation/decomposition (e.g., chemical reactions or deposition), kinetic transfer between different pools/layers/reservoirs (e.g., between the troposphere and the stratosphere) or kinetic partitioning (e.g., between gas and liquid phases). Some of these processes, e.g., sink kinetics, are active throughout the lifecycle of a component, and thereby continuously push concentrations towards log-normality.

Overall, the implications of log-normal concentration distributions span a broad spectrum of potential applications, ranging from data analysis methodologies, sampling strategies, emissions estimation, source apportionment calculations, modelling of chemical fate, estimation of toxic exposure to future climate scenarios^9,25,29,41–52^. Given the general mathematical formulation of the model (Eq. 2), we note that the applicability extends to log-normal concentration distributions also beyond Environmental systems^53,54^.

A specific example, where mechanistic insights may be derived from analysis of concentration distributions is the estimation of lifetimes (τ = 1/*k*, Eq. 1)^55,56^. Our present findings suggest that observation of log-normally distributed concentration data, perhaps especially at remote or receptor sites, may indicate sink kinetics, and may therefore provide insights into the sink rate (Eqs. 4a–b and 5). Another specific situation where concentration distribution analysis is of importance is the common challenge of trend detection in environmental data^11^. For certain time-series data, log-transformation is commonly used prior to statistical analysis^51,57^. The present analysis may provide a physico-chemical motivation for such transformations, potentially contributing to interpretations. A third specific example regards the analysis of ratios of diagnostic markers, which are common tools to assess sources and processes across Earth and Environmental sciences. Examples include ratios of chemical markers and isotope signatures, where the latter commonly are reported as concentration ratios. The ratio of two log-normal random variables is another log-normal variable^58^. We may then predict that if the overall concentrations are log-normal, then, e.g., isotope signatures should also be log-normal, with potential implications for data analysis methodology and interpretation.

Finally, we note that rates in environmental systems often are proportional to concentrations, e.g., reactants. As an example, the sink rate for CO is proportional to the OH concentration (see Theory). Consequently, if the concentration is log-normal, so is the rate. Furthermore, fluxes (e.g., emission or sink fluxes) are often defined as the product of a rate and a concentration (or at least the amount, by some proportional measure). Taken together, this suggests that log-normal concentration distributions may imply log-normal distributions also for rates and/or fluxes for certain systems, with implications for, e.g., emission estimation or box models^47,49,59^.

All-in-all, this paper presents a mechanistic model for how log-normal concentrations may emerge in the environment, which in turn suggests an explanation for the relative abundance.

## Supplementary Information

Supplementary Information.
