# Publisher Correction: Mechanisms for log normal concentration distributions in the environment

**DOI:** 10.1038/s41598-021-97822-2

**Published:** 2021-09-06

**Authors:** August Andersson

**Affiliations:** Department of Environmental Science and the Bolin Centre for Climate Research, Stockholm University, 10691 Stockholm, Sweden

Correction to: *Scientific Reports* 10.1038/s41598-021-96010-6, published online 12 August 2021

The original version of this Article contained an error in the Theory section where Greek symbols did not display correctly.

As a result,

“We may re-write Eq. (1) in the form of a stochastic differential equation (SDE):

2 $$\frac{d[X]}{{dt}} = - (\mu_{k} + \sigma_{k} \eta (t))[X]$$

where μ_k_ is the mean reaction rate and μ_k_ is the magnitude of the stochastic fluctuation. The function μ(t) describes the time-dependency of the random fluctuations (with amplitude 1), which we here assume is independent and identically (i.i.d.) normal distributed.

Since μ(t) fluctuates randomly at each time point, the solutions to Eq. (2) will also fluctuate randomly, and we obtain a different solution (or path) when solving it at different instances.”

now reads:

“We may re-write Eq. (1) in the form of a stochastic differential equation (SDE):

2 $$\frac{d[X]}{{dt}} = - (\mu_{k} + \sigma_{k} \eta (t))[X]$$

where μ_k_ is the mean reaction rate and σ_k_ is the magnitude of the stochastic fluctuation. The function η(t) describes the time-dependency of the random fluctuations (with amplitude 1), which we here assume is independent and identically (i.i.d.) normal distributed.

Since η(t) fluctuates randomly at each time point, the solutions to Eq. (2) will also fluctuate randomly, and we obtain a different solution (or path) when solving it at different instances.”

“It is instructive to examine the temporal evolution of the mean (*m*), which for a log-normal distribution is given by (here, *k* = μ_k_, since the mean of μ(t) is zero)”.

now reads:

“It is instructive to examine the temporal evolution of the mean (*m*), which for a log-normal distribution is given by (here, *k* = μ_k_, since the mean of η(t) is zero)”.

The original Article has been corrected.

