# Supplementary material for: Mechanisms for log normal concentration distributions in the environment
Source: Sci Rep. 2021 Aug 12;11:16418. doi: 10.1038/s41598-021-96010-6 (PMC8360985; doi:10.1038/s41598-021-96010-6)
Supplement: Supplementary file 1 — Supplementary Information. [file 41598_2021_96010_MOESM1_ESM.pdf]

Supplementary Information

Mechanisms for Log Normal Concentrations Distributions in the Environment

August Andersson

Department of Environmental Science and the Bolin Centre for Climate Research

Stockholm University, 10691 Stockholm, Sweden

Contents:

Page 2: S1. A stochastic differential equation for sink kinetics.

Page 7: S2. A stochastic differential equation for formation kinetics.

Page 8: References

S1. A stochastic differential equation for sink kinetics

In this section we show that the concentration distribution, i.e., the probability density distribution (pdf), that describes the variability of a first order sink process with a randomly varying rate is the log-normal distribution. Mathematically, we represent this sink process by a stochastic differential equation (SDE): Eq. (2) in the main manuscript (here reproduced as Eq. (S1) for convenience).

The derivation of the log-normal distribution from this SDE is composed of 5 main steps:

i) Perform a variable change of the SDE, where the multiplicative noise term is replaced by an expression with additive noise, which is more convenient for further analysis.

ii) Find the Fokker-Planck equation (FPE) that corresponds to the SDE with the additive noise term. The FPE is a partial differential equation the describe the time-evolution of the pdf.

iii) Solve the FPE.

iv) Invert the variable transformation from step i), but for the solution of the FPE.

v) Establish the boundary conditions for the solution at t = 0.

There is no mathematical novelty in this derivation, as solutions have been presented for mathematically similar models[1,2]. However, we here express the model for the specific system of pysico/chemical sink kinetics. We aim for an explicit and hopefully transparent presentation, allowing for readers that are not familiar with SDEs to follow the argument. This includes a simplified notation, akin to regular calculus. The resulting partial differential equations are for clarity also solved explicitly.

i). Variable transformation

Eq. (S1) represents a SDE where the random term ($\eta(t)$) is multiplied with the function we want to solve for (X):

$$\frac{d[X]}{dt} = -\big(\mu_k + \sigma_k \eta(t)\big)[X] \qquad \text{(S1)}$$

We assume that η(t)) is identical and independent normal distributed with mean equal to zero and standard deviation equal to one.

In Eq. (S1) we have what is called multiplicative noise: the noise term is multiplied with the variable X. However, the standard formulation of SDEs is based on additive noise. We therefore need to do a variable transformation of the form:

$$Z = \ln([X]) \tag{S2}$$

In regular calculus, we would then compute the differential:

$$dZ = \frac{d[X]}{[X]} \tag{S3}$$

For stochastic calculus, this relation does not apply. Instead, we calculate the differential using Itô's formula.

Itô's formula and variable transformation in SDEs

Consider a general stochastic differential equation of the form:

$$\frac{d[X]}{dt} = A([X],t) + B([X],t)\eta(t) \tag{S4}$$

Where A and B are functions of X and t, while η(t) is the stochastic noise term. Now we want to introduce a variable change Z = f([X]) (e.g., Z = ln ([X])).

Itô's formula gives the following general expression for the differential:

$$d(f([X],t),t) = \left(A([X],t)\frac{\partial f}{\partial X} + \frac{\partial f}{\partial t} + \frac{1}{2}B([X],t)^2\frac{\partial^2 f}{\partial X^2}\right)dt + B([X],t)\frac{\partial f}{\partial X}\eta(t)dt \tag{S5}$$

We have:

$$A(X,t) = -\mu_k[X] \tag{S6a}$$

$$B(X,t) = -\sigma_k[X] \tag{S6b}$$

$$f = ln[\mathrm{X}] \tag{S6c}$$

$$\frac{\partial f}{\partial [X]} = \frac{1}{[X]} \tag{S6d}$$

$$\frac{\partial^2 f}{\partial[X]^2} = -\frac{1}{[X]^2} \tag{S6e}$$

$$\frac{\partial f}{\partial t} = 0 \tag{S6f}$$

Taken together we get:

$$\frac{dZ}{dt} = -\left(\mu_k + \frac{1}{2}\sigma_k^2\right) - \sigma_k \eta(t) \tag{S7}$$

This transformation removes the multiplicative noise term, and replaces it with an expression with additive noise, compare with Eq. (S1).

ii). The Fokker-Planck equation

Now we want to relate this stochastic differential equation to an equation describing the time-evolution of the concentration distribution of Z, i.e., the probability density distribution (P(Z,t)). The equation that describes the evolution of P(Z,t)) is a partial differential equation called the Fokker-Planck equation (FPE). Eq. (S7) is a SDE in a standard form, with an additive noise term, and the corresponding FPE given by:

$$\frac{\partial P(Z,t)}{\partial t} = \frac{\sigma_k^2}{2}\frac{\partial^2 P(Z,t)}{\partial Z^2} + \left(\mu_k + \frac{1}{2}\sigma_k^2\right)\frac{\partial P(Z,t)}{\partial Z} \tag{S8}$$

For a formal derivation of Eq. (S8) from Eq. (S7), see, e.g., Zwanzig[3].

iii). Solving the Fokker Planck equation: Separation of variables

To solve the partial differential equation (S8), we use separation of variables, setting:

$$P(Z,t) = e^{st+qZ} b(Z,t) \tag{S9}$$

The corresponding partial derivatives are:

$$\frac{\partial P(Z,t)}{\partial t} = \left(sb(Z,t) + \frac{\partial b(Z,t)}{\partial t}\right) e^{st+qZ} \tag{S10a}$$

$$\frac{\partial P(Z,t)}{\partial Z} = \left(qb(Z,t) + \frac{\partial b(Z,t)}{\partial Z}\right) e^{st+qZ} \tag{S10b}$$

$$\frac{\partial^2 P(Z,t)}{dZ^2} = \left(q^2 b(Z,t) + 2q\frac{\partial b(Z,t)}{\partial Z} + \frac{\partial^2 b(Z,t)}{\partial Z^2}\right)e^{st+qZ} \tag{S10c}$$

We plug this into the Fokker-Planck equation, (S8):

$$sb(Z,t) + \frac{\partial b(Z,t)}{\partial t} = \frac{\sigma_k^2}{2}\left(q^2 b(Z,t) + 2q\frac{\partial b(Z,t)}{\partial Z} + \frac{\partial^2 b(z,t)}{\partial Z^2}\right) + \left(\mu_k + \frac{1}{2}\sigma_k^2\right)\left(qb(Z,t) + \frac{\partial b(Z,t)}{\partial Z}\right) \tag{S11}$$

We can then define q and s as:

$$0 = \sigma_k^2 q + \frac{1}{2}\sigma_k^2 \rightarrow q = -\frac{\mu_k + \frac{1}{2}\sigma_k^2}{\sigma_k^2} \tag{S12}$$

$$s = \frac{\sigma_k^2}{2}q^2 + \left(\mu_k + \frac{1}{2}\sigma_k^2\right)q = -\frac{\left(\mu_k + \frac{1}{2}\sigma_k^2\right)^2}{2\sigma_k^2} \tag{S13}$$

The Diffusion Equation

Through these definitions, the direct dependencies on b(Z,t) and $\frac{\partial b(Z,t)}{\partial Z}$ in Eq. (S11) are removed, and we have:

$$\frac{\partial b(Z,t)}{\partial t} = \frac{\sigma_k^2}{2}\frac{\partial^2 b(Z,t)}{\partial Z^2} \tag{S14}$$

Which is formally equivalent to the diffusion/heat equation, which we do not solve here, see, e.g., Zwanzig[3]. The solution is a normal distribution:

$$b(Z,t) = \frac{1}{\sqrt{2\pi\sigma_k^2 t}}e^{-\frac{Z^2}{2\sigma_k^2 t}} \tag{S15}$$

Combing Eqs (S9), (S12-S13) and (S15), we have:

$$P(Z,t) = \frac{1}{\sqrt{2\pi\sigma_k^2 t}}e^{-\frac{\left(Z + \mu_k t + \frac{1}{2}\sigma_k^2 t\right)^2}{2\sigma_k^2 t}} \tag{S16}$$

Which is a normal distribution with mean $-\mu_k t - \frac{1}{2}\sigma_k^2 t$ and variance $\sigma_k^2 t$.

iv). Back-transformation

Now we want to invert the variable transformation of Eq. (S2). We emphasize the absence of any stochastic terms, and we have:

$$Z = \ln([X]) \tag{S17}$$

$$dZ = \left|\frac{d[X]}{[X]}\right| \tag{S18}$$

Combining Eqs. (S16 – S18), we obtain the log-normal distribution:

$$P([X],t) = \frac{1}{x\sqrt{2\pi\sigma_k^2 t}} e^{-\frac{\left(\ln([X])+\mu_k t+\frac{1}{2}\sigma_k^2 t\right)^2}{2\sigma_k^2 t}} \tag{S19}$$

v). Boundary Condition: Distribution at t = 0

Finally, we need to establish the distribution of X at the boundary condition t = 0. For mathematical convenience we assume that it is log-normal (with parameters μ_0 and σ_0). The product distribution of two log-normally distributed random variables, (C = A·B), is another log-normal distribution[4]. The parameters for this log-normal product distribution are:

$$\mu_C = \mu_A + \mu_B \tag{S20a}$$

$$\sigma_C^2 = \sigma_A^2 + \sigma_B^2 \tag{S20b}$$

We arrive at:

$$P([X],t) = \frac{1}{x\sqrt{2\pi\left(\sigma_0^2+\sigma_k^2 t\right)}} e^{-\frac{\left(\ln([X])-\mu_0+\left(\mu_k+\frac{1}{2}\sigma_k^2\right)t\right)^2}{2\left(\sigma_0^2+\sigma_k^2 t\right)}} \tag{S21}$$

S2. A stochastic differential equation for formation kinetics

In the main manuscript, the formulation of the kinetics model is focused on sink or loss processes. But what about processes involving formation or production?

Consider Eq. (2) from the main manuscript (Eq (S1) reprinted for convenience:

$$\frac{d[X]}{dt} = -\left(\mu_k + \sigma_k \eta(t)\right)[X] \tag{S22}$$

which describes the breakdown of a component X. Now consider that as a result of this reaction, that a product Y is formed (e.g., CO_2 is formed by the breakdown of CO by OH): what would be the concentration distribution for Y?

The formation kinetics for Y may be written as (removing the minus sign in Eq. (S22)):

$$\frac{d[Y]}{dt} = \left(\mu_k + \sigma_k \eta(t)\right)[X] \tag{S23}$$

Combining Eqs. (S22) and (S23):

$$\frac{d[Y]}{dt} = -\frac{d[X]}{dt} \tag{S24}$$

Using the Stratanovich integral for stochastic calculus, we can solve for Z(t) as:

$$[Y] = \int -\frac{d[X]}{dt} dt = -[X] + [X(0)] \tag{S25}$$

Since X is a log-normally distributed stochastic variable we conclude that Y may also be a log normally distributed stochastic variable.

References

1. Black, F., Scholes, M. The Pricing of Options and Corporate Liabilities. *J. Polit. Econ.* **81**, 637-645 (1973).

2. Crow, E. L.; Shimiziu, K. Lognormal distributions: Theory and Applications. ISBN: 0367580276. (CRC Press., 1988).

3. Zwanzig, R. Non-equilibrium statistical mechanics. ISBN: 978-0195140187 (Oxford University Press., 2001**)**.

4. Springer, M., D. The algebra of random variables. ISBN: 0471014060 (Wiley, 1979).
